# Experimental Realization of a Multiscroll Chaotic Oscillator with Optimal Maximum Lyapunov Exponent

**DOI:** 10.1155/2014/303614

**Published:** 2014-04-16

**Authors:** Esteban Tlelo-Cuautle, Ana Dalia Pano-Azucena, Victor Hugo Carbajal-Gomez, Mauro Sanchez-Sanchez

**Affiliations:** ^1^INAOE, Department of Electronics, 72480 Puebla, MEX, Mexico; ^2^Universidad del Papaloapan, Loma Bonita, 68400 Oaxaca, MEX, Mexico

## Abstract

Nowadays, different kinds of experimental realizations of chaotic oscillators have been already presented in the literature. However, those realizations do not consider the value of the maximum Lyapunov exponent, which gives a quantitative measure of the grade of unpredictability of chaotic systems. That way, this paper shows the experimental realization of an optimized multiscroll chaotic oscillator based on saturated function series. First, from the mathematical description having four coefficients (*a, b, c, d_1_*), an optimization evolutionary algorithm varies them to maximize the value of the positive Lyapunov exponent. Second, a realization of those optimized coefficients using operational amplifiers is given. Herein *a, b, c, d_1_* are implemented with precision potentiometers to tune up to four decimals of the coefficients having the range between 0.0001 and 1.0000. Finally, experimental results of the phase-space portraits for generating from 2 to 10 scrolls are listed to show that their associated value for the optimal maximum Lyapunov exponent increases by increasing the number of scrolls, thus guaranteeing a more complex chaotic behavior.

## 1. Introduction

Chaos is a multidisciplinary research area that is being ubiquitous in all engineering areas, such as electronics, control, communication, and security. Basically, engineers are interested in the analysis, realization, and application of chaos [1–3]. For instance, in electronics we are interested in the realization of chaotic oscillators, in which mathematical descriptions have three main characteristics: a chaotic oscillator is sensitive to initial conditions, it is nonperiodic, and it is deterministic, because the coefficients of its mathematical description are known [4]. Further, a measure for quantifying chaos in dynamical systems is by computing the value of the Lyapunov exponents, from which one gets information on their grade of unpredictability [5]. That way, in continuous-time chaotic oscillators the number of state variables determines the number of Lyapunov exponents, so that for a third order dynamical system, the three Lyapunov exponents for generating chaos should be negative, zero, and positive. Similarly, higher order dynamical systems should possess at least one positive Lyapunov exponent to guarantee chaotic regime. The positive Lyapunov exponent is known as maximum Lyapunov exponent as well.

In current literature, one can find realizations of chaotic oscillators using different kinds of amplifiers and in some cases using integrated circuit technology, as listed in [6]. For instance, the authors in [7] have already implemented Chua's circuit to generate multiscroll chaotic attractors by using commercially available current-feedback operational amplifiers (CFOAs). For that multiscroll chaotic oscillator, its positive Lyapunov exponent was evaluated to verify that it was in chaotic regime. The novelty of that work was the introduction of a new circuit for realizing its piecewise-linear (PWL) function using CFOAs, where the parameters were computed systematically to implement symmetrical or nonsymmetrical PWL functions. However, in that work and in the very majority of the already introduced circuit realizations [6], the value of the maximum Lyapunov exponent (MLE) is just evaluated but not optimized. Henceforth, this paper shows, by experiments, that multiscroll chaotic oscillators with optimal MLE can have a more complex chaotic behavior. In this manner, the optimization task to compute the MLE of a chaotic oscillator based on saturated nonlinear function (SNLF) series is performed herein by applying the differential evolution algorithm already introduced in [8], and which basically searches for the optimal values of the coefficients of the mathematical description of the multiscroll chaotic oscillator. Further, the feasible solutions for the coefficients providing high values of the MLE are implemented using commercially available operational amplifiers to demonstrate that the optimized values have a more complex chaotic behavior than by using traditional values, as the ones given in [4].

Section 2 describes the generalities of the differential evolution algorithm applied in [8] and the procedure to compute the Lyapunov exponents [5].  Section 3 describes the mathematical model of the multiscroll chaotic oscillator that is based on SNLF series [4], and the simulation of attractors generating from 2 to 18 scrolls is given using MATLAB and the circuit simulator SPICE.  Section 3 also introduces the circuit realization using operational amplifiers.  Section 4 demonstrates the realizability of the multiscroll chaotic oscillator having high values of the (positive) maximum Lyapunov exponent (MLE). The experiments show that the chaotic behavior is more complex when the MLE is optimized. As a result, the optimized chaotic oscillators can be synchronized [9, 10] to enhance applications like designing secure communication systems [4]. Finally, Section 5 summarizes the conclusions.

## 2. Optimization Method and Lyapunov Exponent

### 2.1. Differential Evolution Algorithm

Heuristic optimization methods such as evolutionary algorithms are used to optimize problems having very big search spaces. For instance, in this paper the case of study is a multiscroll chaotic oscillator (described in the following section), which has four coefficients *a*, *b*, *c*, *d*_1_. If the range of each coefficient is between 0 and 1, and by considering 4 decimals, then the search space, as mentioned in [8], is very big; that is, it is 16 × 10^16^, thus justifying the use of heuristics.

One pretty good heuristic method was introduced by Storn and Price in 1995; it was called differential evolution, which is quite useful for global optimization over continuous spaces [11]. The main characteristics of differential evolution algorithm for optimizing the MLE in chaotic oscillators are as follows. It converges faster than other evolutionary algorithms because the crossover operation is performed by selecting 3 fathers, and it depends on few parameters than, for example, genetic algorithms, thus augmenting its performance. Differential evolution algorithm keeps a population of candidate solutions that are recombined and mutated to create children that are evaluated by a fitness function, and then the best ones are selected, surviving for the next generation until generating feasible solutions selected from the Pareto front. The pseudo code and implementation details can be found in [8].

### 2.2. Lyapunov Exponent

Lyapunov exponents are asymptotic measures characterizing the contraction or growing rate of small perturbations on the solutions of a dynamical system, and they provide quantitative measures on the sensitivity response of a chaotic system to small changes in the initial conditions [5, 12]. Lyapunov exponents can be evaluated from the following expression:

(1) $$\begin{array}{c} \lambda \left(x_{0};r\right)=\underset{N\to \infty }{\lim}\frac{1}{N}\overset{N-1}{\underset{n=0}{\sum }}\ln\left|\frac{\partial f\left(x;r\right)}{\partial x}\right|, \end{array}$$

where *f*(*x*; *r*) function is discretized in time, *x*_0_, and *r* parameters. If *λ*(*x*_0_; *r*) > 0, then one can say that the dynamical system is in chaotic regime.

Considering an *n*-dimensional dynamical system of the form:

(2) $$\begin{array}{c} \dot{x}=f\left(x\right)\;t>0, \end{array}$$

where *x* and *f* are *n*-dimentional vectors, then a system evolving from (2) in an *n*-dimensional space will present a Lyapunov exponent that depends on the initial condition *x*_0_. On the other hand, by using the Jacobian matrix one can determine the *n*-Lyapunov exponents [12], by evolving the small perturbations to a space trajectory described by [8]

(3) $$\begin{array}{c} \dot{y}=\frac{\partial f}{\partial x}\left(x\left(t\right)\right)y=J\left(x\left(t\right)\right)y,\\ y\left(t\right)=Y\left(t\right)y\left(0\right). \end{array}$$

## 3. Modeling the Multiscroll Chaotic Oscillator 

In this paper, the case of study is the multiscroll chaotic oscillator based on saturated nonlinear function (SNLF) series [4], which is described by

(4) x˙=y,y˙=z,z˙=−ax−by−cz+d1f(x;k,h,p,q),

where *k* > 2; *f*(*x*; *k*, *h*, *p*, *q*) = SNLF series; *x*, *y*, *z* = state variables; *a*, *b*, *c*, *d*_1_ = coefficients with values between 0.0001 and 1.0000. This mathematical model is solved by numerical integration methods, where its matrix description form is given by

(5) $$\begin{array}{c} \left[\begin{array}{c} \dot{x}\\ \dot{y}\\ \dot{z} \end{array}\right]=\left[\begin{array}{ccc} 0 & 1 & 0\\ 0 & 0 & 1\\ -a & -b & -c \end{array}\right]\left[\begin{array}{c} x\\ y\\ z \end{array}\right]+\left[\begin{array}{c} 0\\ 0\\ d_{1}f\left(x;\propto ,k,h,p,q\right) \end{array}\right]. \end{array}$$

In (5) the SNLF series is scaled by ∝ to accommodate the magnitudes to the ones provided by commercially available electronic circuits. The SNLF is described by

(6) $$\begin{array}{c} f\left(x;\propto ,k,h,p,q\right)=\overset{q}{\underset{i=-p}{\sum }}f_{i}\left(x;h,k\right). \end{array}$$

This format to describe the SNLF is called piecewise-linear (PWL) approach, where the equivalent description for (6) is given by

(7) f(x;∝,k,h,p,q)={(2q+1)kx>qh+∝k∝(x−ih)+2ik|x−ih|≤∝, −p≤i≤q(2i+1)kih+∝<x<(i+1)h−∝,−p≤i≤q−1−(2p+1)kx<−ph−∝.

**Figure 1:**
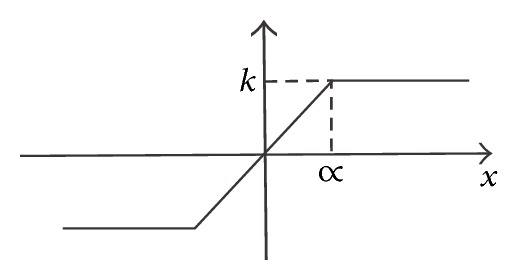
Basic SNLF cell with two saturated levels.

Figure 1 shows the SNLF for generating two scrolls. Its PWL description is given by

(8) $$\begin{array}{c} f\left(x\right)=\{\begin{array}{cc} \propto & x>k\\ s\left(x\right) & -\propto \leq x\leq \propto \\ -\propto & x<-k. \end{array} \end{array}$$

Table 1 shows SNLFs and their corresponding PWL descriptions to generate from 3 to 6 scrolls. As one sees, the number of scrolls is the number of saturated plateaus. For generating more scrolls one should follow the same process from Table 1, basically by increasing the number of saturated levels.

In (5) one can set the values: *a* = *b* = *c* = *d*_1_ = 0.7, *k* = 1,  ∝ = 16.5*e* − 3, *s* = 60.606 and *h*_1_≅1; then, the other values for the PWL functions are shown in Table 2.

**Figure 2:**
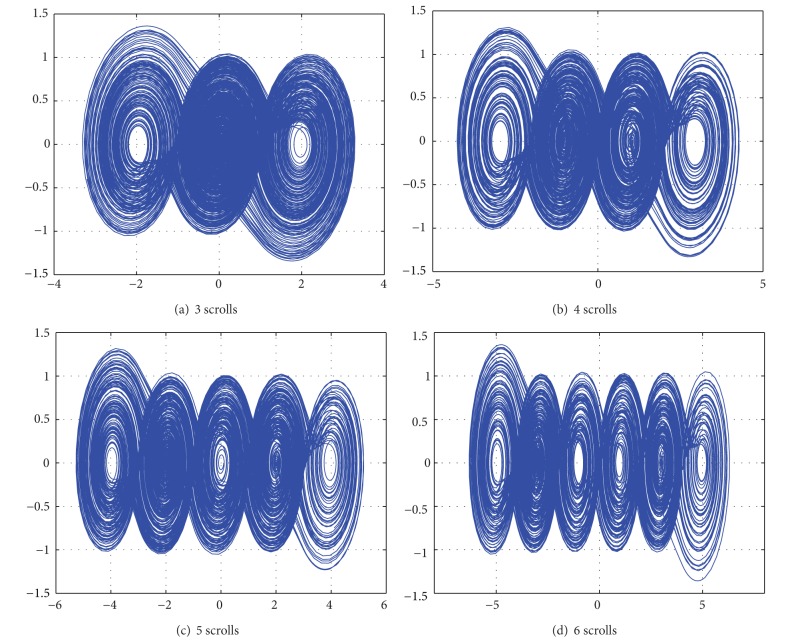
Phase space portraits for generating from 3 to 6 scrolls using MATLAB.

According to Tables 1 and 2, one can obtain the phase space portraits for generating from 3 to 6 scrolls, as shown in Figure 2.

**Figure 3:**
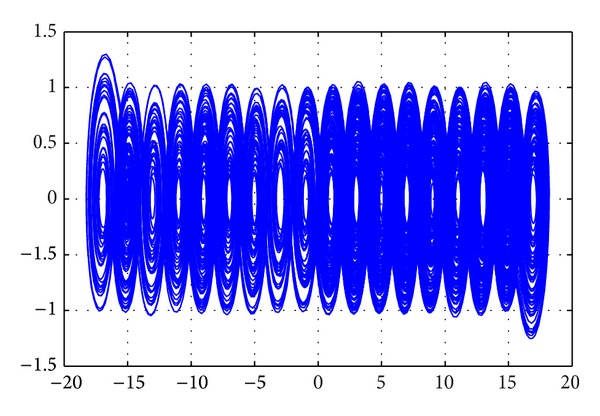
Attractor with 18 scrolls.

To generate 18 scrolls, the PWL function is decomposed as shown in the following expression and the phase space portrait is shown in Figure 3:

(9) f(x;∝,k,h,p,q)={(2q+1k)x>(qh+∝)s(x−8h)+16k(qh−∝)≤x≤(qh+∝)s(x−7h)+14k(q−1)h−∝≤x≤(q−1)h+∝s(x−6h)+12k(q−2)h−∝≤x≤(q−2)h+∝s(x−5h)+10k(q−3)h−∝≤x≤(q−3)h+∝s(x−4h)+8k(q−4)h−∝≤x≤(q−4)h+∝s(x−3h)+6k(q−5)h−∝≤x≤(q−5)h+∝s(x−2h)+4k(q−6)h−∝≤x≤(q−6)h+∝s(x−h)+2kh−∝≤x≤h+∝15k(q−1)h+∝≤x≤(qh−∝)13k(q−2)h+∝≤x≤(q−1)h−∝11k(q−3)h+∝≤x≤(q−2)h−∝9k(q−4)h+∝≤x≤(q−3)h−∝7k(q−5)h+∝≤x≤(q−4)h−∝5k(q−6)h+∝≤x≤(q−5)h−∝3kh+∝≤x≤(q−6)h−∝k∝<x<h−∝sx−∝≤x≤∝s(x+8h)−16k(−qh−∝)≤x≤(−qh+∝)s(x+7h)−14k(−q+1)h−∝≤x≤(−q+1)h+∝s(x+6h)−12k(−q+2)h−∝≤x≤(−q+2)h+∝s(x+5h)−10k(−q+3)h−∝≤x≤(−q+3)h+∝s(x+4h)−8k(−q+4)h−∝≤x≤(−q+4)h+∝s(x+3h)−6k(−q+5)h−∝≤x≤(−q+5)h+∝s(x+2h)−4k(−q+6)h−∝≤x≤(−q+6)h+∝s(x+h)−2k−h−∝≤x≤−h+∝−15k(−qh+∝)≤x≤(−q+1)h−∝−13k(−q+1)h+∝≤x≤(−q+2)h−∝−11k(−q+2)h+∝≤x≤(−q+3)h−∝−9k(−q+3)h+∝≤x≤(−q+4)h−∝−7k(−q+4)h+∝≤x≤(−q+5)h−∝−5k(−q+5)h+∝≤x≤(−q+6)h−∝−3k(−q+6)h+∝≤x≤h−∝−k−h+∝<x<−∝−(2p+1)kx<(−qh−∝).

**Figure 4:**
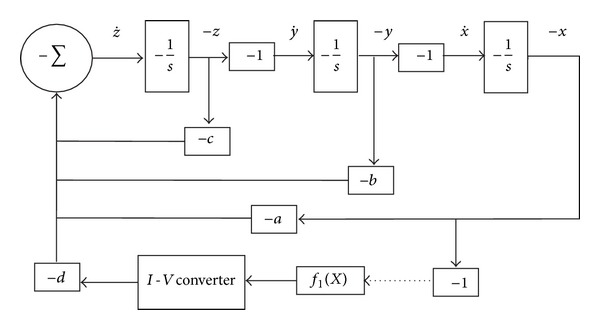
Block diagram of (5).

**Figure 5:**
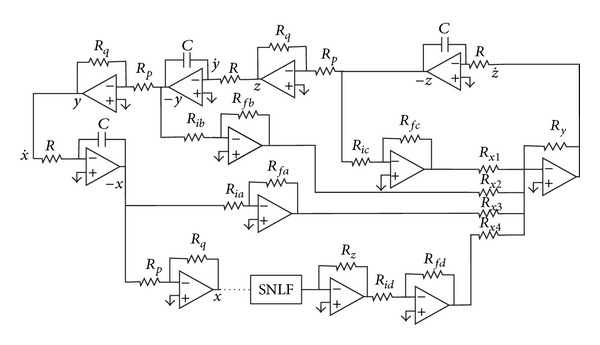
Realization of Figure 4 using opamps.

The chaotic oscillator described by (5) can be designed as shown in Figure 4, while its realization using operational amplifiers (opamps) is given in Figure 5.

**Figure 6:**
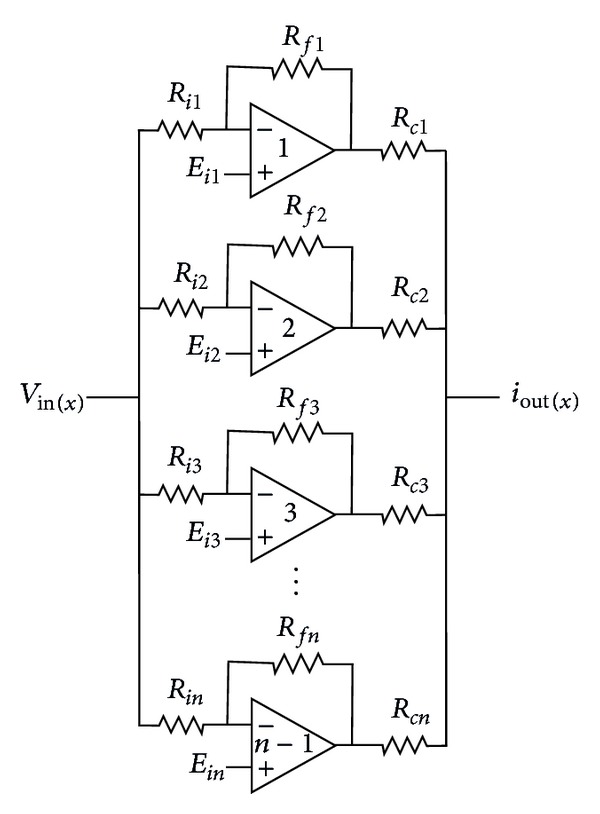
Realization of the SNLF using operational amplifiers.

The SNLF can be implemented as shown in Figure 6, where the number of opamps equals the number of scrolls to be generated minus 1. The term *E*_*i*_ represents a shifted voltage, and the other parameters of the PWL functions are evaluated by

(10) $$\begin{array}{c} k=R_{ix}*I_{\mathrm{sat}}\;\propto =\frac{R_{i}\left|V_{\mathrm{sat}}\right|}{R_{f}}\;s=\frac{k}{\propto }\\ I_{\mathrm{sat}}=\frac{V_{\mathrm{sat}}}{R_{c}}\;h≅E_{i}. \end{array}$$

**Figure 7:**
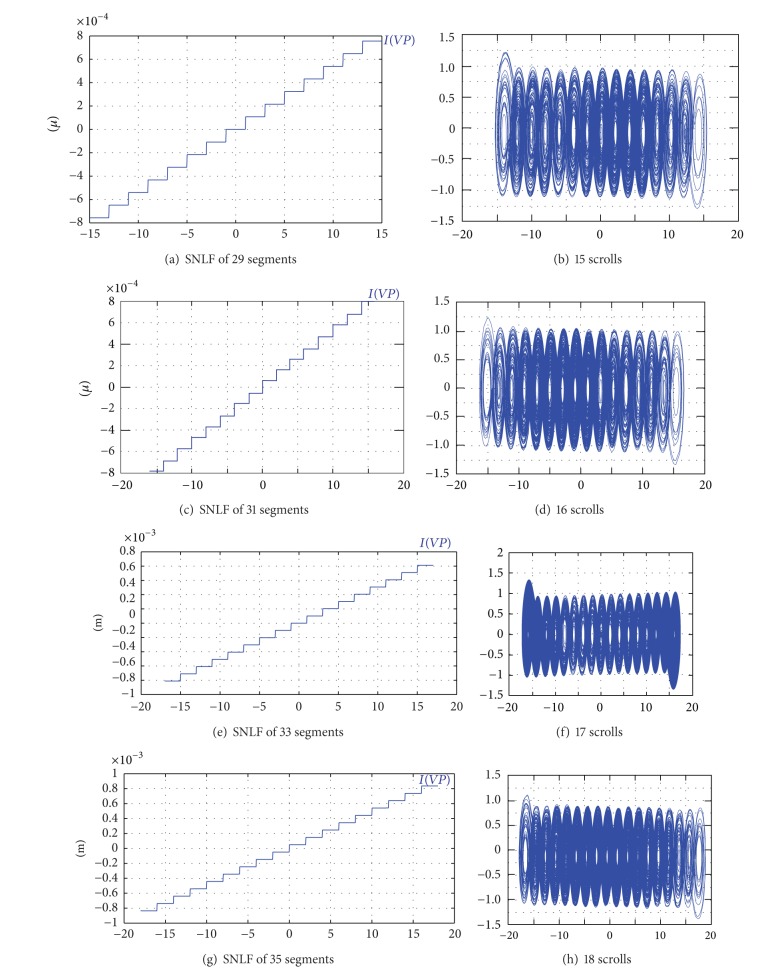
SNLF for generating 15 to 18 scrolls using SPICE.

Simulating (5) in the circuit simulator SPICE, using the model for the commercially available opamp TL081, and the diagrams shown in Figures 5 and 6, one can generate the attractors from 2 to 18 scrolls using the circuit element values shown in Table 3 and Figure 7. Using (5), (6), and (10), the parameter values are *a* = *b* = *c* = *d*_1_ = 0.7, *k* = 1, ∝ = 16.5*e* − 3, *s* = 60.606, *h*_1_≅1, *I*_sat⁡_ = 100 *μ*A, *R*_*ix*_ = 10 kΩ, *C* = 1 *μ*F, *R* = 1 MegΩ, *R*_*ia*_ = *R*_*ib*_ = *R*_*ic*_ = *R*_*id*_ = 10 kΩ, *R*_*fa*_ = *R*_*fb*_ = *R*_*fc*_ = *R*_*fd*_ = 7 kΩ, *R*_*i*_ = *R*_*f*_, *F* ≈ 162 mHz, and *V*_sat⁡_ = ±16.

## 4. Experimental Results

**Figure 8:**
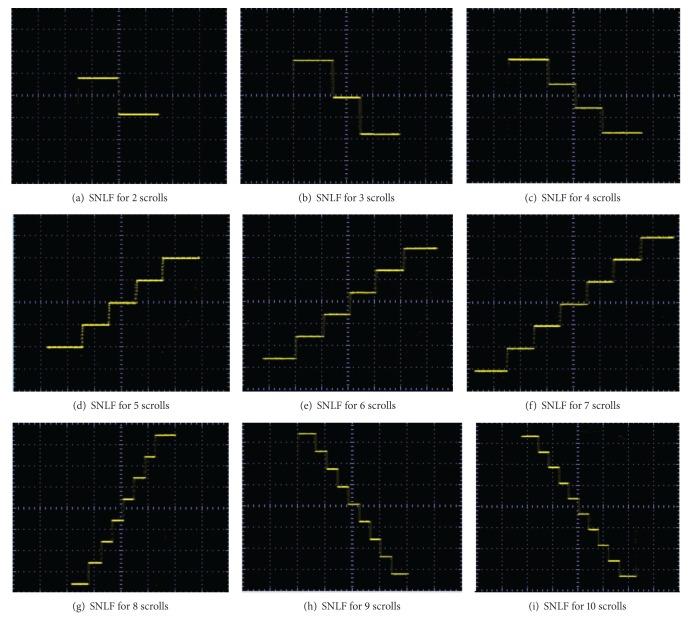
Experimental results of the SNLF for generating from 2 to 10 scrolls.

The realization of the SNLF shown in Figure 6 is performed by using commercially available operational amplifiers like the TL081. Experimental results using the values of Table 3 lead us to Figure 8, for generating from 2 to 10 scrolls.

The SNLFs shown in Figure 8 are used to generate multiscroll chaotic attractors. In this case, the coefficients in (5) are optimized by applying the differential evolution algorithm described in [8]. For generating from 2 to 10 scrolls, Tables 4 and 5 list the optimized coefficient values for *a*, *b*, *c*, *d*_1_ having a high value for the (positive) maximum Lyapunov exponent (MLE). In all cases, *k* = 10 and *h* = 2.

As one sees in Table 4, using the traditional values of *a* = *b* = *c* = *d*_1_ = 0.7, the value of MLE is small compared to the other optimized values.  Table 5 summarizes several optimized combinations for generating from 5 to 10 scrolls.

**Figure 9:**
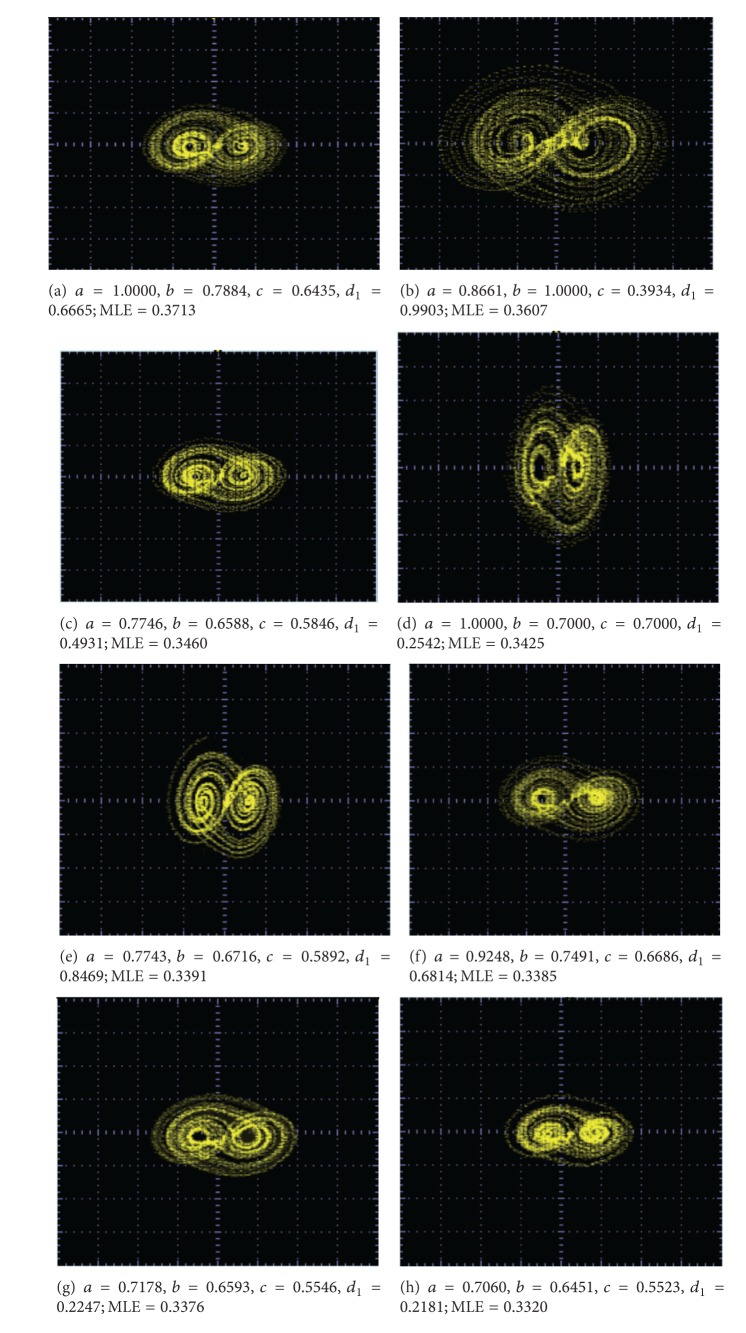
Optimized MLE for generating 2 scrolls.

**Figure 10:**
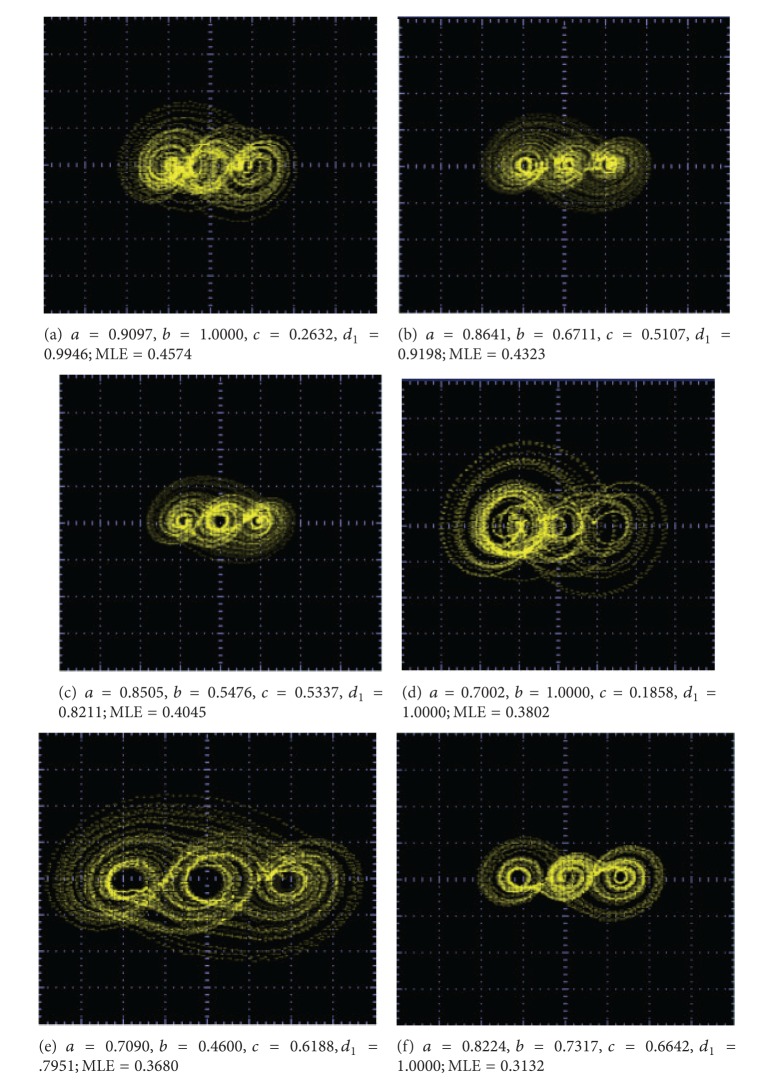
Optimized MLE for generating 3 scrolls.

**Figure 11:**
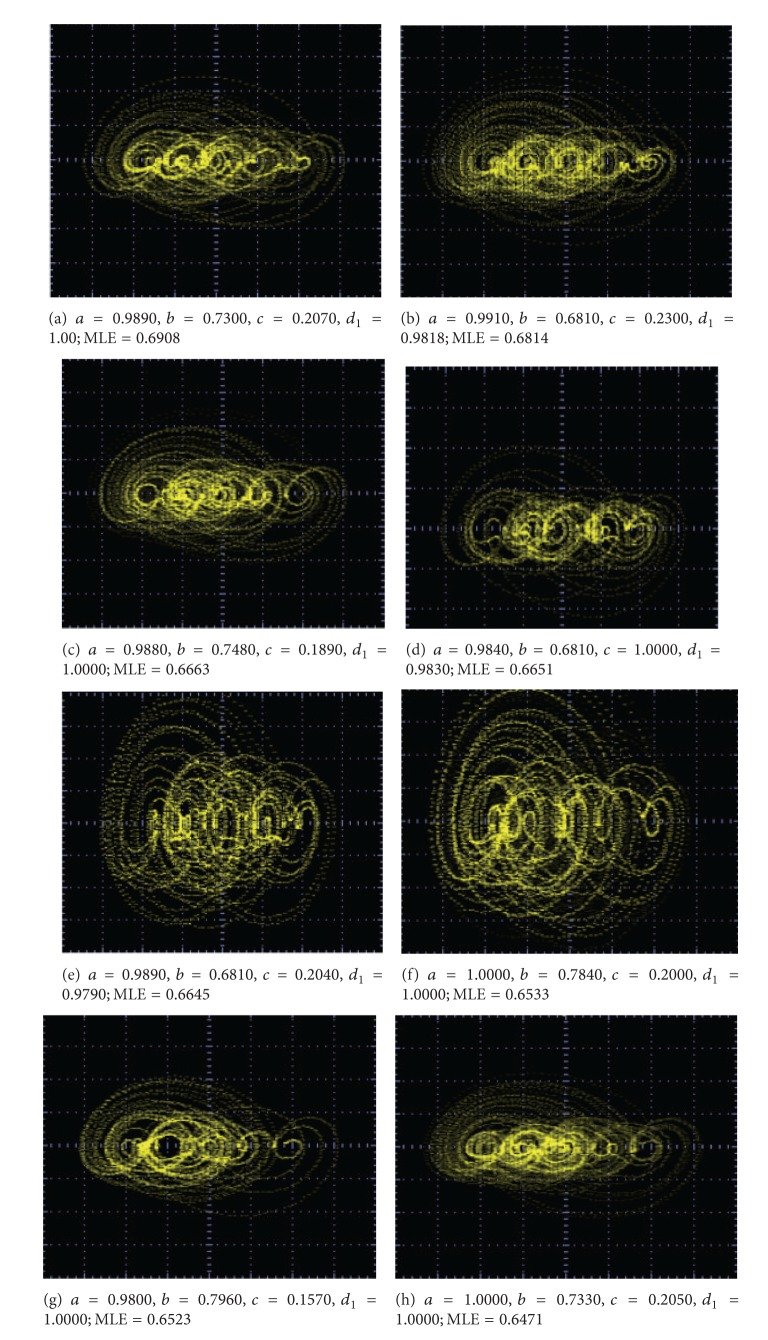
Optimized MLE for generating 5 scrolls.

**Figure 12:**
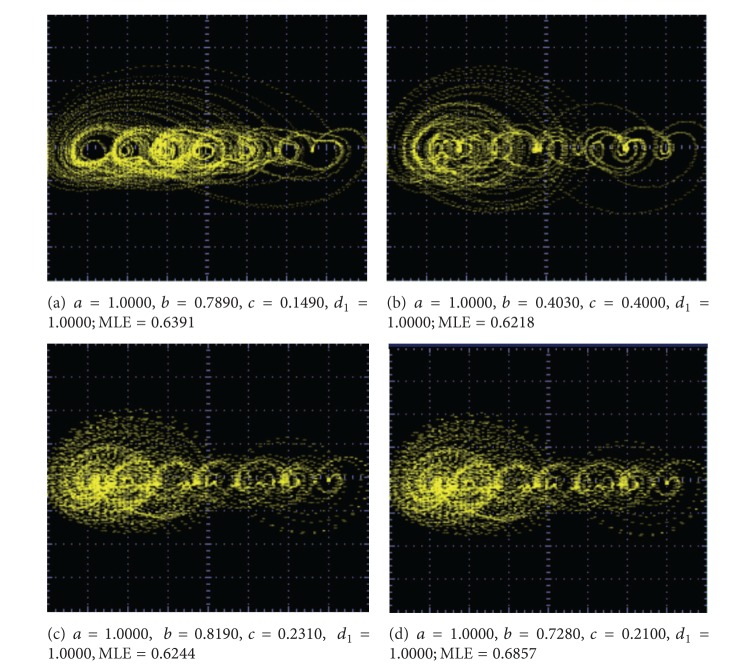
Optimized MLE for generating 7 scrolls.

**Figure 13:**
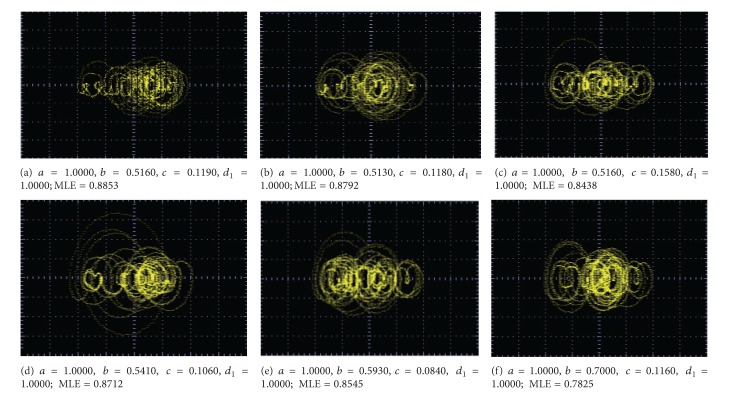
Optimized MLE for generating 10 scrolls.

For generating two scrolls experimentally, the values of the circuit elements in Figures 5 and 6 are *C* = 1 nF, *R* = 1 MegΩ, *R*_*ia*_ = *R*_*ib*_ = *R*_*ic*_ = *R*_*id*_ = 10 kΩ, *R*_*i*_ = *R*_*f*_; the operational amplifiers where biased with *V*_sat⁡_ = ± 16 V and ±18 V. To adjust the values of the coefficients *a*, *b*, *c*, *d*_1_ with four decimals, linear precision potentiometers were used, for example, *R*_*fa*_, *R*_*fb*_, *R*_*fc*_, and *R*_*fd*_ in Figure 5. Several experimental results for some optimized values of *a*, *b*, *c*, *d*_1_ are shown in Figures 9–13.

From these experimental results, one can infer that the value of the MLE increases just by increasing the number of scrolls. More important is that the chaotic behavior becomes more complex when the MLE is optimized. That way, not only chaotic synchronized systems [9, 10] can become be more robust but also other complex systems like hyperchaotic systems [13] and with more directions [14] (2D, 3D, etc.) it may be improved using optimized multiscroll chaotic oscillators.

## 5. Conclusion

The experimental realization of a multiscroll chaotic oscillator based on saturated nonlinear function (SNLF) series has been presented. Its (positive) maximum Lyapunov exponent was optimized and the feasible solutions (combinations) of the coefficient values for *a*, *b*, *c*, *d*_1_ were ranked and listed in Tables 4 and 5. The multiscroll chaotic oscillator was implemented with the commercially available operational amplifier TL081, and the values of the circuit elements were listed along with the values for the SNLF in Table 3. In addition, SPICE simulations were presented in Figure 7 to generate from 15 to 18 scrolls, and experimental results were given for generating 2, 3, 5, 7, and 10 scrolls in Figures 9, 10, 11, 12, and 13, respectively.

As a conclusion, the optimized coefficients *a*, *b*, *c*, *d*_1_ were realized with precision potentiometers to generate the attractor, which showed more complex chaotic behavior as the maximum Lyapunov exponent increases. Another interesting conclusion is that the value of the maximum Lyapunov exponent is higher when the number of scrolls is increased, meaning that the more scrolls are generated, the more the guarantee of chaotic regime.

## Figures and Tables

**Table 1:** Description of the SNLF for generating from 3 to 6 scrolls.

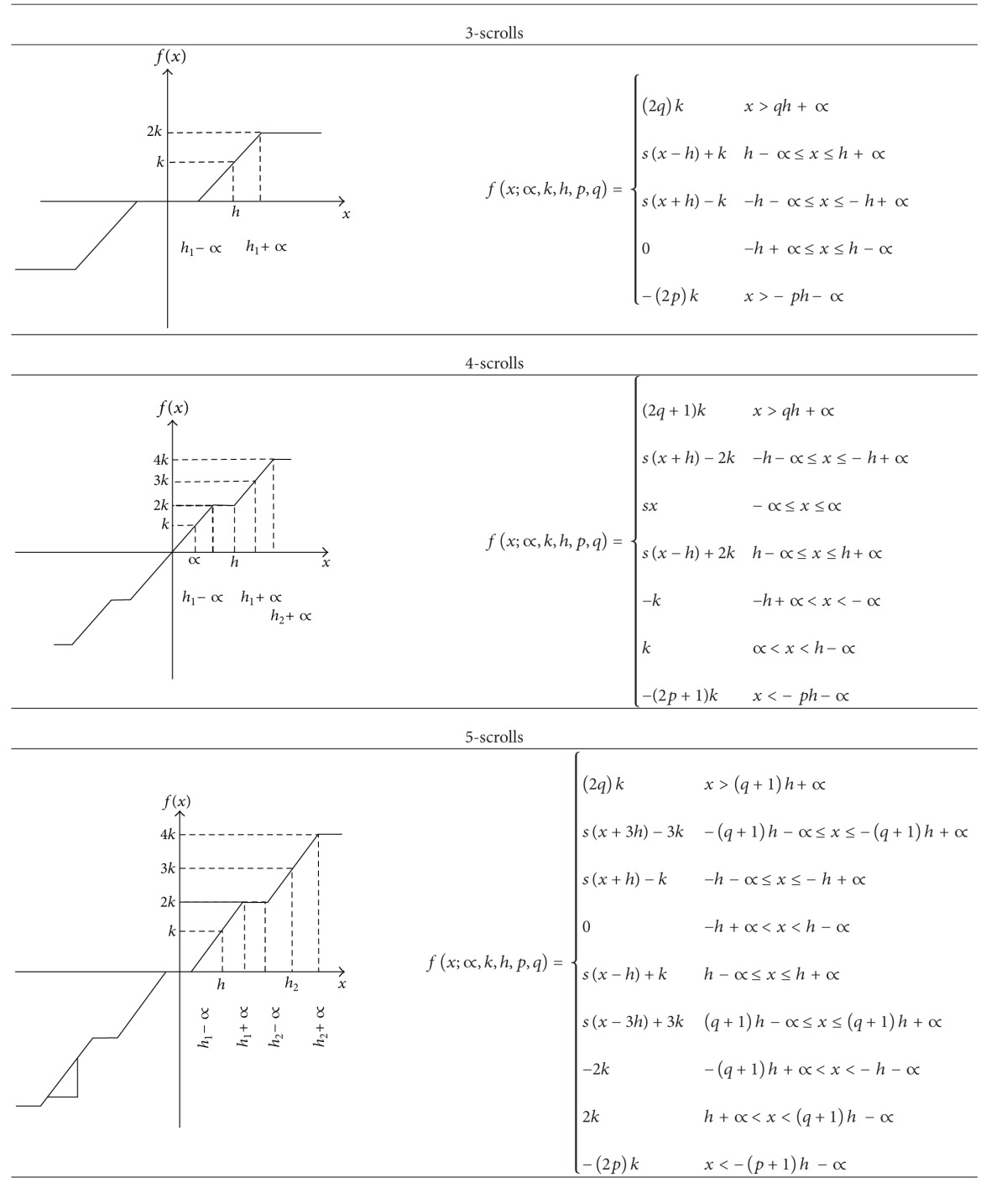 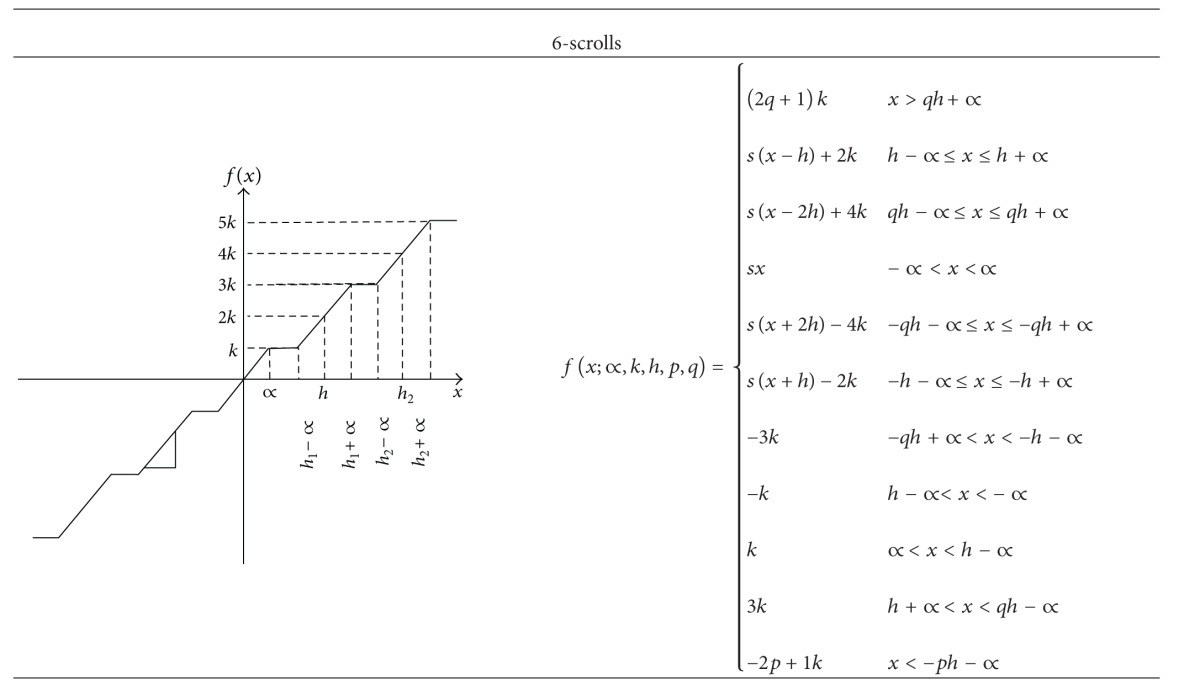

**Table 2:** Values for generating from 3 to 18 scrolls.

Scrolls	Values for (5) and (6)	Simulation time (s)
3	*K* = 1, *∝* = 16.5 m, *s* = 60.606, *h* = 1, *p* = 1, *q* = 1	6000
4	*K* = 1, ∝ = 16.5 m, *s* = 60.606, *h* = 1, *p* = 1, *q* = 1	8000
5	*K* = 1, ∝ = 16.5 m, *s* = 60.606, *h* = 1, *p* = 2, *q* = 2	10000
6	*K* = 1, ∝ = 16.5 m, *s* = 60.606, *h* = 1, *p* = 2, *q* = 2	12000
7	*K* = 1, ∝ = 16.5 m, *s* = 60.606, *h* = 1, *p* = 3, *q* = 3	14000
8	*K* = 1, ∝ = 16.5 m, *s* = 60.606, *h* = 1, *p* = 3, *q* = 3	16000
9	*K* = 1, ∝ = 16.5 m, *s* = 60.606, *h* = 1, *p* = 4, *q* = 4	18000
10	*K* = 1, ∝ = 16.5 m, *s* = 60.606, *h* = 1, *p* = 4, *q* = 4	20000
11	*K* = 1, ∝ = 16.5 m, *s* = 60.606, *h* = 1, *p* = 5, *q* = 5	22000
12	*K* = 1, ∝ = 16.5 m, *s* = 60.606, *h* = 1, *p* = 5, *q* = 5	24000
13	*K* = 1, ∝ = 16.5 m, *s* = 60.606, *h* = 1, *p* = 6, *q* = 6	26000
14	*K* = 1, ∝ = 16.5 m, *s* = 60.606, *h* = 1, *p* = 6, *q* = 6	28000
15	*K* = 1, ∝ = 16.5 m, *s* = 60.606, *h* = 1, *p* = 7, *q* = 7	30000
16	*K* = 1, ∝ = 16.5 m, *s* = 60.606, *h* = 1, *p* = 7, *q* = 7	32000
17	*K* = 1, ∝ = 16.5 m, *s* = 60.606, *h* = 1, *p* = 8, *q* = 8	34000
18	*K* = 1, ∝ = 16.5 m, *s* = 60.606, *h* = 1, *p* = 8, *q* = 8	36000

**Table 3:** Values for generating from 2 to 18 scrolls using SPICE.

Scrolls	Values			Time
2	*K* = 1, ∝ = 16.5 m, *s* = 60.606, *h* = 1, Isat = 100 uA	Rc1 = 165 KΩ, Ri1 = 1 kΩ, Rf1 = 1 MegΩ	Ei1 = 0 V	300 s

3	*K* = 1, ∝ = 16.5 m, *s* = 60.606, *h* = 1, Isat = 100 uA	Rc1 = Rc2 = 165 KΩ, Ri1 = Ri2 = 1 kΩ, Rf1 = Rf2 = 1 MegΩ	Ei1 = +1 V, Ei2 = −1 V	500 s

4	*K* = 1, ∝ = 16.5 m, *s* = 60.606, *h* = 1, Isat = 100 uA	Rc1 = *⋯* = Rc3 = 165 KΩ, Ri1 = *⋯* = Ri3 = 1 kΩ Rf1 = *⋯* =Rf3 = 1 MegΩ	Ei1 = +2 V, Ei2 = −2 V, Ei3 = 0 V	700 s

5	*K* = 1, ∝ = 16.5 m, *s* = 60.606, *h* = 1, Isat = 100 uA	Rc1 = *⋯* = Rc4 = 165 KΩ, Ri1 = *⋯* = Ri4 = 1 kΩ, Rf1 = *⋯* = Rf4 = 1 MegΩ	Ei1 = +1 V, Ei2 = −1 V, Ei3 = +3 V, Ei4 = −3 V	900 s

6	*K* = 1, ∝ = 16.5 m, *s* = 60.606, *h* = 1, Isat = 100 uA	Rc1 = *⋯* = Rc5 = 165 KΩ, Ri1 = *⋯* = Ri5 = 1 KΩ, Rf1 = *⋯* = Rf5 = 1 MegΩ	Ei1 = +2 V, Ei2 = −2 V, Ei3 = +4 V, Ei4 = −4 V,Ei5 = 0 V	1000 s

7	*K* = 1, ∝ = 16.5 m, *s* = 60.606, *h* = 1, Isat = 100 uA	Rc1 = *⋯* = Rc6 = 165 KΩ, Ri1 = *⋯* = Ri6 = 1 kΩ, Rf1 = *⋯* = Rf6 = 1 MegΩ	Ei1 = +5 V, Ei2 = −5 V, Ei3 = +1 V, Ei4 = −1 V, Ei5 = +3 V, Ei6 = −3 V	3000 s

8	*K* = 1 , ∝ = 16.5 m, *s* = 60.606, *h* = 1, Isat = 100 uA	Rc1 = *⋯* = Rc7 = 165 KΩ, Ri1 = *⋯* = Ri7 = 1 kΩ, Rf1 = *⋯* = Rf7 = 1 MegΩ	Ei1 = +4 V, Ei2 = −4 V, Ei3 = +2 V, Ei4 = −2 V, Ei5 = +6 V, Ei6 = −6 V, Ei7 = 0 V.	5000 s

9	*K* = 1, ∝ = 16.5 m, *s* = 60.606, *h* = 1, Isat = 100 uA	Rc1 = *⋯* = Rc8 = 165 KΩ, Ri1 = *⋯* = Ri8 = 1 kΩRf1 = *⋯* = Rf8 = 1 MegΩ	Ei1 = +3 V, Ei2 = −3 V, Ei3 = +1 V, Ei4 = −1 V, Ei5 = +5 V, Ei6 = −5 V, Ei7 = +7 V, Ei8 = −7 V	6000 s

10	*K* = 1, ∝ = 16.5 m, *s* = 60.606, *h* = 1, Isat = 100 uA	Rc1 = *⋯* = Rc9 = 165 KΩ, Ri1 = *⋯* = Ri9 = 1 kΩ Rf1 = *⋯* = Rf9 = 1 MegΩ	Ei1 = +8 V, Ei2 = −8 V, Ei3 = +4 V, Ei4 = −4 V, Ei5 = +2 V, Ei6 = −2 V, Ei7 = +6 V, Ei8 = −6 V, Ei9 = 0 V	7000 s

11	*K* = 1, ∝ = 16.5 m, *s* = 60.606, *h* = 1, Isat = 100 uA	Rc1 = *⋯* = Rc10 = 165 KΩ, Ri1 = *⋯* = Ri10 = 1 kΩ Rf1 = *⋯* = Rf10 = 1 MegΩ	Ei1 = +9 V, Ei2 = −9 V, Ei3 = +7 V, Ei4 = −7 V, Ei5 = +5 V, Ei6 = −5 V, Ei7 = +3 V, Ei8 = −3 V, Ei9 = +1 V, Ei10 = −1 V	9000 s

12	*K* = 1, ∝ = 16.5 m, *s* = 60.606, *h* = 1, Isat = 100 uA	Rc1 = *⋯* = Rc11 = 165 KΩ, Ri1 = *⋯* = Ri11 = 1 kΩ, Rf1 = *⋯* = Rf12 = 1 MegΩ	Ei1 = +10 V, Ei2 = −10 V, Ei3 = +8 V, Ei4 = −8 V, Ei5 = +6 V, Ei6 = −6 V, Ei7 = +4 V, Ei8 = −4 V, Ei9 = 2 V, Ei10 = −2 V, Ei11 = 0 V	12000 s

13	*K* = 1, ∝ = 16.5 m, *s* = 60.606, *h* = 1, Isat = 100 uA	Rc1 = *⋯* = Rc12 = 165 KΩ, Ri1 = *⋯* = Ri12 = 1 kΩ, Rf1 = *⋯* = Rf12 = 1 MegΩ	Ei1 = +9 V, Ei2 = −9 V, Ei3 = +7 V, Ei4 = −7 V, Ei5 = +5 V, Ei6 = −5 V, Ei7 = +3 V, Ei8 = −3 V, Ei9 = 1 V, Ei10 = −1 V, Ei11 = 11 V, Ei12 = −11 V	13000 s

14	*K* = 1, ∝ = 16.5 m, *s* = 60.606, *h* = 1, Isat = 100 uA	Rc1 = *⋯* = Rc13 = 165 KΩ, Ri1 = *⋯* = Ri13 = 1 kΩ, Rf1 = *⋯* = Rf13 = 1 MegΩ	Ei1 = 8 V, Ei2 = −8 V, Ei3 = 4 V, Ei4 = −4 V, Ei5 = +2 V, Ei6 = −2 V, Ei7 = +6 V, Ei8 = −6 V, Ei9 = 0 V, Ei10 = 10 V, Ei11 = −10 V, Ei12 = 12 V, Ei13 = −12 V	14000 s

15	*K* = 1, ∝ = 16.5 m, *s* = 60.606, *h* = 1, Isat = 100 uA	Rc1 = *⋯* = Rc14 = 165 KΩ, Ri1 = *⋯* = Ri14 = 1 kΩ, Rf1 = *⋯* = Rf14 = 1 MegΩ	Ei1 = +9 V, Ei2 = −9 V, Ei3 = +7 V, Ei4 = −7 V, Ei5 = +5 V, Ei6 = −5 V, Ei7 = +3 V, Ei8 = −3 V, Ei9 = 1 V, Ei10 = −1 V, Ei11 = 11 V, Ei12 = −11 V, Ei13 = 13 V, Ei14 = −13 V	15000 s

16	*K* = 1, ∝ = 16.5 m, *s* = 60.606, *h* = 1, Isat = 100 uA	Rc1 = *⋯* = Rc15 = 165 KΩ, Ri1 = *⋯* = Ri15 = 1 kΩ, Rf1 = *⋯* = Rf15 = 1 MegΩ	Ei1 = +8 V, Ei2 = −8 V, Ei3 = +4 V, Ei4 = −4 V, Ei5 = +2 V, Ei6 = −2 V, Ei7 = +6 V, Ei8 = −6 V, Ei9 = 0 V, Ei10 = 10 V, Ei11 = 10 V, Ei12 = 12 V, Ei13 = −12 V, Ei14 = 14 V, Ei15 = −14 V	17000 s

17	*K* = 1, ∝ = 16.5 m, *s* = 60.606, *h* = 1, Isat = 100 uA	Rc1 = *⋯* = Rc16 = 165 KΩ, Ri1 = *⋯* = Ri16 = 1 kΩ, Rf1 = *⋯* = Rf16 = 1 MegΩ	Ei1 = +9 V, Ei2 = −9 V, Ei3 = +7 V, Ei4 = −7 V, Ei5 = +5 V, Ei6 = −5 V, Ei7 = +3 V, Ei8 = −3 V, Ei9 = 1 V, Ei10 = −1 V, Ei11 = 11 V, Ei12 = −11 V, Ei13 = 13 V, Ei14 = −13 V, Ei15 = 15 V, Ei16 = −15 V	18000 s

18	*K* = 1, ∝ = 16.5 m, *s* = 60.606, *h* = 1, Isat = 100 uA	Rc1 = *⋯* = Rc17 = 165 KΩ, Ri1 = *⋯* = Ri17 = 1 kΩ, Rf1 = *⋯* = Rf17 = 1 MegΩ	Ei1 = +8 V, Ei2 = −8 V, Ei3 = +4 V, Ei4 = −4, Ei5 = +2 V, Ei6 = −2 V, Ei7 = +6 V, Ei8 = −6 V, Ei9 = 0 V, Ei10 = 10 V, Ei11 = −10 V, Ei12 = 12 V, Ei13 = −12 V, Ei14 = 14 V, Ei15 = −14 V, Ei16 = 16 V, Ei17 = −16 V	20000 s

**Table 4:** Optimized maximum Lyapunov exponent for generating 2 scrolls.

Coefficients *a*, *b*, *c*, *d*_1_	Maximum Lyapunov exponent
*a* = 1.0000, *b* = 1.0000, *c* = 0.4997, *d*_1_ = 1.0000	0.3761
*a* = 1.0000, *b* = 0.7884, *c* = 0.6435, *d*_1_ = 0.6665	0.3713
*a* = 0.8661, *b* = 1.0000, *c* = 0.3934, *d*_1_ = 0.9903	0.3607
*a* = 0.7746, *b* = 0.6588, *c* = 0.5846, *d*_1_ = 0.4931	0.3460
*a* = 1.0000, *b* = 0.7000, *c* = 0.6780, *d*_1_ = 0.1069	0.3437
*a* = 1.0000, *b* = 0.7000, *c* = 0.7000, *d*_1_ = 0.2542	0.3425
*a* = 0.7743, *b* = 0.6716, *c* = 0.5892, *d*_1_ = 0.8469	0.3391
*a* = 0.9248, *b* = 0.7491, *c* = 0.6686, *d*_1_ = 0.6814	0.3385
*a* = 0.7178, *b* = 0.6593, *c* = 0.5546, *d*_1_ = 0.2247	0.3376
*a* = 0.7060, *b* = 0.6451, *c* = 0.5523, *d*_1_ = 0.2181	0.3320
*a* = 0.7000, *b* = 0.7000, *c* = 0.7000, *d*_1_ = 0.7000	0.2658

**Table 5:** Optimized MLE for 5 to 10 scrolls and their (*a*,  *b*,  *c*,  *d*_1_) coefficient values.

5 scrolls	6 scrolls	7 scrolls
0.6919	0.72	0.7313
(1.0000, 0.7250, 0.2250, 1.000)	(1.0000, 0.6750, 0.2100, 1.000)	(1.0000, 0.6430, 0.1580, 1.0000)
0.6914	0.7107	0.7182
(0.9880, 0.7140, 0.2050, 1.000)	(1.0000, 0.6870, 0.2160, 1.000)	(1.0000, 0.6110, 0.1390, 0.9750)
0.6908	0.706	0.7174
(0.9890, 0.7300, 0.2070, 1.0000)	(1.0000, 0.6920, 0.1700, 1.000)	(1.0000, 0.6410, 0.1390, 1.0000)
0.6814	0.6904	0.6952
(0.9910, 0.6810, 0.2300, 0.9810)	(1.0000, 0.6870, 0.1830, 1.000)	(1.0000, 0.5320, 0.1920, 0.9960)
0.6663	0.6764	0.6857
(0.9880, 0.7480, 0.1890, 1.0000)	(0.9940, 0.6510, 0.2320, 0.9860)	(1.0000, 0.7280, 0.2100, 1.0000)
0.6651	0.6758	0.6391
(0.9840, 0.6810, 0.2270, 0.9830)	(1.0000, 0.7530, 0.1740, 1.000)	(1.0000, 0.7890, 0.1490, 1.0000)
0.6645	0.6741	0.6244
(0.9890, 0.6810, 0.2040, 0.9790)	(1.0000, 0.7060, 0.1850, 1.000)	(1.0000, 0.8190, 0.2310, 1.0000)
0.6533	0.6245	0.6218
(1.0000, 0.7840, 0.2000, 1.0000)	(0.9780, 0.7690, 0.2270, 1.000)	(1.0000, 0.4030, 0.4000, 1.0000)
0.6523	0.5871	0.505
(0.9800, 0.7960, 0.1570, 1.0000)	(1.0000, 0.6140, 0.3190, 1.000)	(0.9470, 1.0000, 0.2220, 0.9110)
0.6471	0.563	0.4263
(1.0000, 0.7330, 0.2050, 1.0000)	(0.9670, 0.8230, 0.3380, 0.955)	(0.9050, 0.6100, 0.6180, 0.8590)

8 scrolls	9 scrolls	10 scrolls

0.8412	0.8654	0.8853
(1.0000, 0.5690, 0.1360, 0.9970)	(0.9960, 0.5370, 0.1450, 0.998)	(1.0000, 0.5160, 0.1190, 1.0000)
0.8382	0.8595	0.8792
(1.0000, 0.5750, 0.1290, 0.9980)	(1.0000, 0.5800, 0.0960, 1.000)	(1.0000, 0.5130, 0.1180, 1.0000)
0.8208	0.8563	0.8438
(1.0000, 0.6120, 0.1210, 0.9980)	(0.9990, 0.530, 0.1320, 0.9980)	(1.0000, 0.5160, 0.1580, 1.0000)
0.8471	0.8503	0.8712
(1.0000, 0.5880, 0.1280, 1.0000)	(1.0000, 0.5440, 0.1150, 1.000)	(1.0000, 0.5410, 0.1060, 1.0000)
0.8458	0.877	0.8545
(1.0000, 0.5880, 0.1190, 1.0000)	(1.000, 0.5020, 0.1430, 0.9970)	(1.0000, 0.5930, 0.0840, 1.0000)
0.8407	0.8595	0.7825
(0.9800, 0.5720, 0.1270, 1.0000)	(1.0000, 0.5560, 0.103, 1.0000)	(1.0000, 0.7000, 0.1160, 1.0000)
